# VBA: A Probabilistic Treatment of Nonlinear Models for Neurobiological and Behavioural Data

**DOI:** 10.1371/journal.pcbi.1003441

**Published:** 2014-01-23

**Authors:** Jean Daunizeau, Vincent Adam, Lionel Rigoux

**Affiliations:** 1 Brain and Spine Institute, Paris, France; 2 Wellcome Trust Centre for Neuroimaging, University College London, London, United Kingdom; 3 Gatsby computational neuroscience Unit, University College London, London, United Kingdom; UCSD, United States of America

## Abstract

This work is in line with an on-going effort tending toward a computational (quantitative and refutable) understanding of human neuro-cognitive processes. Many sophisticated models for behavioural and neurobiological data have flourished during the past decade. Most of these models are partly unspecified (i.e. they have unknown parameters) and nonlinear. This makes them difficult to peer with a formal statistical data analysis framework. In turn, this compromises the reproducibility of model-based empirical studies. This work exposes a software toolbox that provides generic, efficient and robust probabilistic solutions to the three problems of model-based analysis of empirical data: (i) data simulation, (ii) parameter estimation/model selection, and (iii) experimental design optimization.

This is a *PLOS Computational Biology* Software Article

## Introduction

Spectrum diseases in psychiatry, such as schizophrenia or depression, display profound heterogeneity with regard to the underlying pathophysiological mechanisms, requiring the development of models that can infer subject-specific mechanisms from neurophysiological and/or behavioural data [1]–[3]. Developing quantitative approaches that can do this will require merging expert knowledge on neurobiology, biophysical generation of neuroimaging signals, cognitive psychology, and statistical data analysis, to mention but a few. Recall that most cerebral mechanisms can be described at two levels of abstraction:

- The cognitive or functional level is concerned with the information processing that is needed to explain behavioural measurements (e.g., choices, reaction times) or subjective reports (e.g., emotions, thoughts).
- The neurobiological or physiological level is related to the neurobiological substrate of the system. Imaging neuroscience or neuroimaging (e.g., electroencephalography/magnetoencephalography or EEG/MEG, functional Magnetic Resonance Imaging or fMRI) is capable of observing (non-invasively) certain biophysical characteristics of this biological substrate.

These typically map to two classes of models, i.e. (i) formal models of perception, learning and decision making that predict behavioural responses, and (ii) biophysically realistic models that describe how electrophysiological activity propagate through neural networks. The issue with such models is that they are based upon mechanisms that are usually both hidden (they are not directly accessible from experimental data) and nonlinear (this is the curse of realism). As a consequence, one requires sophisticated statistical approaches that can deal efficiently with parameter estimation and model selection (given experimental data). If only, these are necessary to capture the inter-individual variability of neurophysiological and behavioural responses. More generally, such schemes would embed the models into the data analysis and act as a “mathematical microscope” that is capable of unravelling mechanisms, which are hidden deep within experimental data [4].

This article describes a (matlab) software toolbox that is designed to perform such model-based analyses of neuroimaging and behavioural data. Essentially, it consists of a probabilistic model inversion scheme that borrows from disciplines such as inverse problems, statistical physics and machine learning. More precisely, the toolbox implements a variational Bayesian approach (VBA) that can deal with a very general class of generative models, which we describe below. In brief, VBA address the following issues:

- performing efficient and robust parameter estimation on nonlinear models;
- providing quantitative diagnostics of model fitting (including summary statistics that can be used for model comparison);
- optimizing the experimental design in the aim of maximizing the statistical power of model-based data analysis;
- assessing the results reproducibility at the group level (e.g., between-groups and between-conditions model comparisons).

In addition, the toolbox gathers a growing number of established and novel biophysical and cognitive models, which capture a broad range of phenomena. Among these are deterministic and stochastic variants of dynamic causal models for fMRI data (see, e.g. [5] for a recent review) and bayesian models for human learning and decision making [6]–[7]. Importantly, VBA includes diagnostic analyses that can be used directly to refine such models, i.e. to account for yet unpredicted features of experimental data (cf. Volterra decompositions). We will demonstrate this below. Our ambition is twofold: (i) to disseminate models and methods that serve experimental purposes, and (ii) to provide a flexible platform, which modellers can easily contribute to.

This paper is organized as follows.

In the next section, we will describe the design and implementation of the toolbox. In particular, we will describe the class of generative models that the VBA toolbox can handle, expose the main aspects of its algorithmic treatment, and summarize the organization of the code. In section “Results”, we will demonstrate its capabilities through analyses of deposited test data. In section “Availability and future directions”, we will discuss limitations and on-going developments of this work.

## Design and Implementation

**Figure 1:**
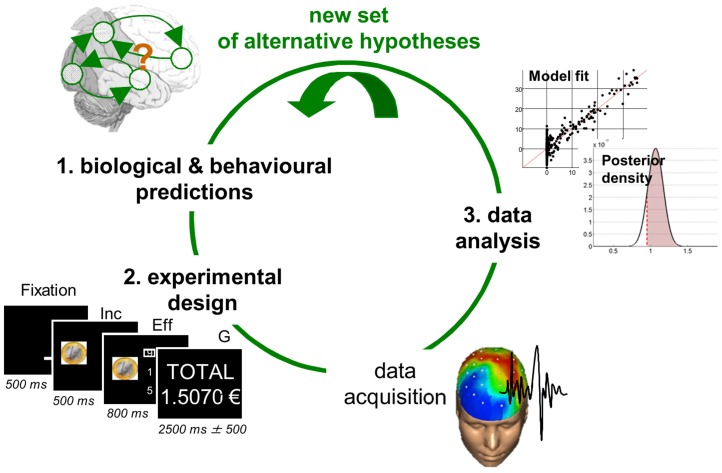
The experimental cycle. The experimental cycle summarizes the interaction between modelling, experimental work and statistical data analysis. One starts with new competing hypotheses about a system of interest. These are then embodied into a set of candidate models that are to be compared with each other given empirical data. One then designs an experiment that is maximally discriminative with respect to the candidate models. Data acquisition and analysis then proceed, the conclusion of which serves to generate a new set of competing hypotheses, etc… Adapted from [14].

In brief, the toolbox furnishes solutions to the three canonical problems of model-based data analysis, which relate to the experimental cycle (cf. Figure 1). One starts with a set of competing hypotheses, e.g.: is the incentive value discounted (or not) by the amount of cognitive effort that is required to obtain the reward? This question will eventually be framed in terms of a model comparison (e.g., model 1 -with effort discounting- versus model 2 -without effort discounting-). First, one has to be able to predict behavioural and/or biological signals from the candidate models. This simply means simulating, e.g. people's choices under model 1 and 2. Second, one may want to optimize the experimental design, in the aim of best discriminating the candidate models. In our example, this naturally involves manipulating both the reward that is at stake and the amount of effort. However, there might be an optimal manipulation of these two factors, such that models 1 and 2 yield radically different predictions. Third, one proceeds to parameter estimation and/or model selection, given the acquired experimental data. Here, statistical inference follows the variational Bayesian treatment of the candidate models (see below).

Data analysis results can now serve to identify a new set of competing hypotheses, which then triggers a new experimental cycle…

The toolbox is designed so that data simulation, parameter estimation, model selection and design optimization can be handled automatically, given standardized information about the model (see below).

### Stochastic nonlinear state-space models

Any Bayesian data analysis relies upon a generative model, i.e. a probabilistic description of the mechanisms by which observed data are generated. Such descriptions can go from simple static linear models to nonlinear dynamical models in continuous time. This toolbox does not invert any generative model: it has been developed to deal with stochastic nonlinear state-space models. As we will see below, this class of generative models grandfathers most models used in the literature. It is defined by a joint probability distribution over the following set of variables:

- *y*: the 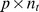 data time-series
- *x*: the 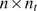 hidden states time-series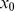: the 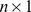 initial conditions of the system
- *u*: the 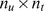 inputs time-series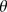: the 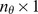 evolution parameters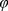: the 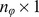 observation parameters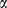: the state noise precision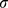: the measurement noise precision

These variables are assumed to obey the following evolution and observation equations:
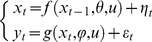
(1)where 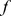 (resp. 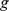) is the evolution (resp. observation) mapping, and 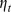 (resp. 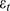) is the state (resp. measurement) noise. As we will see later, continuous dynamical systems can, in principle, be reduced to Equation 1 (cf. Text S3 in the supplementary information). Equation 1 is then augmented with Gaussian prior assumptions regarding the statistical behaviour of initial conditions, evolution/observation parameters and state/measurement noise:
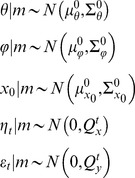
(2)where 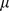 (resp. 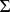) denotes the mean (resp. covariance matrix) of the Gaussian density. Setting the prior variance of a given variable to zero simply means fixing its value to its prior mean. In addition, we use Gamma priors on precision hyperparameters:
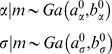
(3)where *a* (resp. *b*) denotes the shape (resp. rate) parameter of the Gamma distribution, which control the first two moments of the Gamma density, as follows: 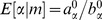, and 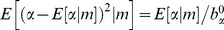. Given priors in Equations 2–3, the first line of Equation 1 induces a (so-called Markovian) prior density on the trajectory of hidden state *x*. Similarly, the second line of Equation 1 now yields a likelihood function, which measures how plausible is any value that experimental measurement *y* can take, under the assumptions (Equations 1–3) of the generative model *m*. The class of generative models that the toolbox handles is in fact slightly more general than can be inferred from Equation 1. In particular, the observation mapping can be modified to deal with categorical (e.g. multinomial) data. Equation 1 defines a (potentially nonlinear) state-space model, which grand-fathers many generative models used in the statistics literature. We will come back to this later on. At this point, suffices to say that one's model can be defined simply in terms of the evolution and observation functions. This means that data simulation, parameter estimation, model selection and design optimization only require the specification of these two functions.

### VB approach to parameter estimation and model selection

Inverting the above generative model *m* means (i) approximating the conditional density 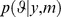 of unknown variables 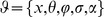 given the set of sampled measurements *y* and (ii) quantifying the model evidence 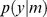. Nonlinearities in the generative model eschew exact analytical solutions to this problem, which is finessed using variational approaches that rest on optimizing a free-energy lower bound 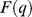 to the model evidence, with respect to an approximate conditional density 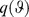 [8]:
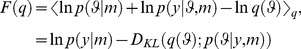
(4)where 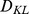 is the Kullback-Leibler divergence and the expectation 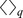 is taken under 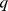. From Equation 4, maximizing the functional 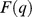 with respect to 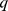 minimizes the Kullback-Leibler divergence between 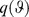 and the exact posterior 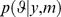. This decomposition is complete in the sense that if 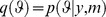, then 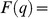

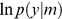
. This means that the free energy 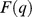 can serve as an analytical approximation to the log model evidence.

**Figure 2:**
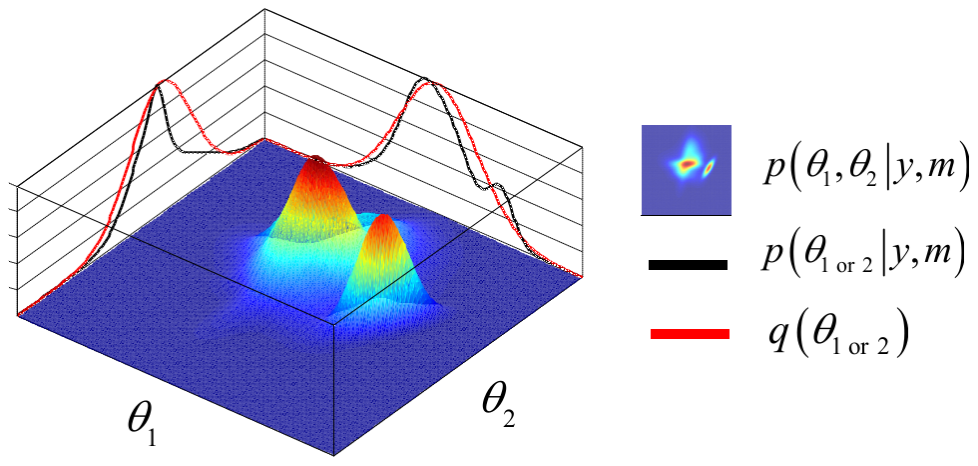
The mean-field/Laplace approximation. The variational Bayesian approach furnishes an approximation to the marginal posterior densities of subsets of unknown model parameters 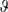. Here, the 2D landscape depicts a (true) joint posterior density 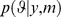 and the two black lines are the subsequent marginal posterior densities of 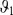 and 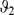, respectively. The mean-field approximation basically describes the joint posterior density as the product of the two marginal densities (black profiles). In turn, stochastic dependencies between parameter subsets are replaced by deterministic dependencies between their posterior sufficient statistics. The Laplace approximation further assumes that the marginal densities can be described by Gaussian densities (red profiles).

Here, the iterative maximization of free energy proceeds under the Laplace approximation, where the approximate posterior 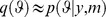 is assumed to have a Gaussian form (except for the precision hyperparameters 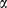 and 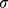, which have Gamma posterior densities; cf. Figure 2). Thus, the variational Bayesian (VB) updates reduce to a regularized Gauss-Newton optimization scheme [9]. This dramatically decreases the computational complexity of the scheme. The second-order moments of the approximate posterior densities are then simply related to the curvature of local cost functions:
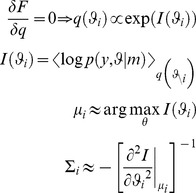
(5)where 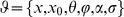 is the set of all unknown model variables, which is partitioned into subsets 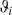 and 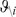 (cf. “mean-field” assumption). Here, the notation “
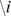
” refers to the complement of the subset indexed by *i*. We refer to [9] for computational details about the VB algorithm. Note that the VB update of the hidden states is very similar in form to a Kalman filter/smoother [10]. More precisely, the scheme derives an approximation to the lagged posterior density 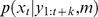, where *k* is the lag (see [11] for the derivation of the lagged forward pass). This lag can be chosen arbitrarily (see below), which allows one to infer on hidden states, whose changes impact observed data a few time samples later in time (e.g. due to some form of convolution operation). The main effect of increasing the lag is to average across more data points when deriving the hidden states, hence improving the precision (and the temporal smoothness) of the estimate. The ensuing computational cost scales with 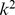.

In brief, the core of the toolbox consists of a generic VB treatment of the above class of generative models. Given experimental data *y*, system's input *u* (if any), evolution/observation mappings and priors, it recovers an approximation to both the posterior density on unknown variables and the model evidence (which is used for model comparison). Practical guidance on the software implementation can be found on this wiki page: http://code.google.com/p/mbb-vb-toolbox/. In particular, we have implemented a lot of examples and demonstration scripts, in the aim of accelerating users' learning (cf. “getting started” section of the wiki pages: http://code.google.com/p/mbb-vb-toolbox/wiki/getting_started).

### Optimization of the experimental design

Optimizing the design in the context of, e.g., experimental psychology studies, amounts to identifying the subset of conditions and stimuli (*u*) that yields the highest statistical power, under a variety of practical constraints. From a modelling perspective, this essentially requires predicting experimental data (*y*) under models' assumptions (*m*), including potential confounds that may mask the effects of interest. Design optimization can become a difficult issue whenever the impact of experimental factors onto measurements (through the model) is non-trivial and/or uncertain (cf. unknown model parameters). This motivates the use of automatic design optimization.

**Figure 3:**
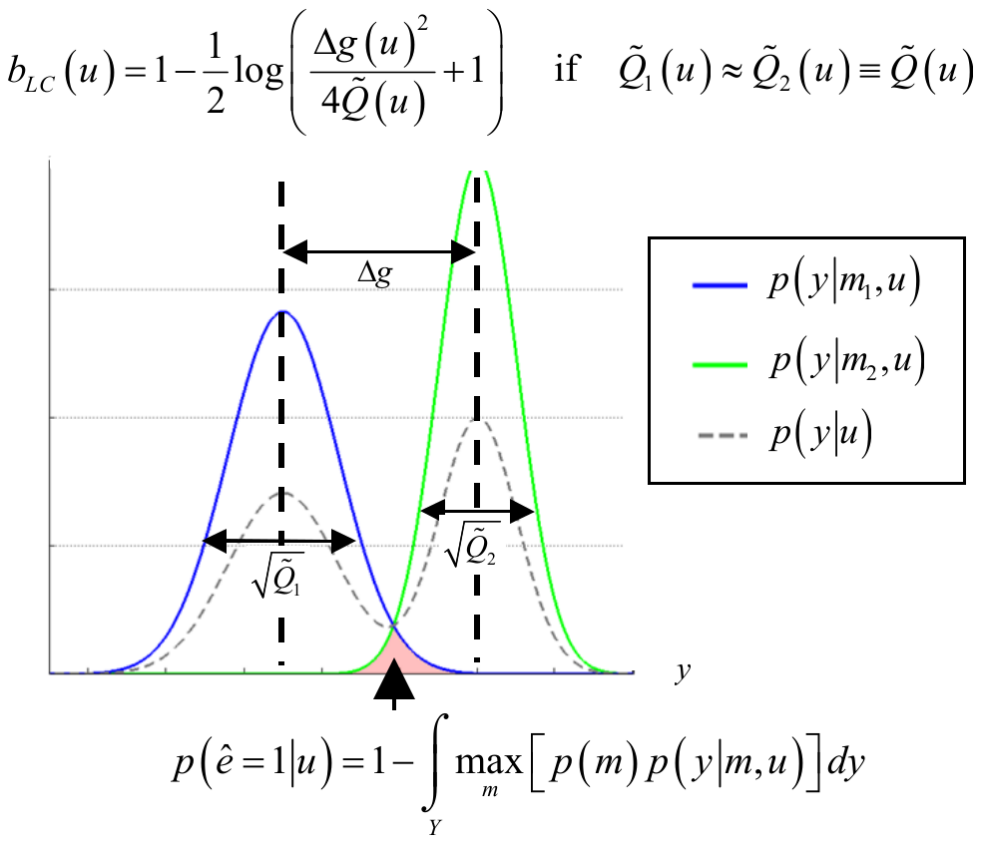
Selection error rate and the Laplace-Chernoff risk. The (univariate) prior predictive density of two generative models 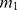 (blue) and 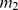 (green) are plotted as a function of data *y*, given an arbitrary design *u*. The dashed grey line shows the marginal predictive density 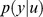 that captures the probabilistic prediction of the whole comparison set 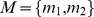. The area under the curve (red) measures the model selection error rate 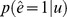, which depends upon the discriminability between the two prior predictive density 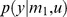 and 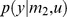. This is precisely what the Laplace-Chernoff risk 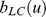 is a measure of. Adapted from [14].

The toolbox can handle two classes of problems, namely optimizing the system's input *u* with respect to either parameter estimation or model selection. These two problems correspond to two different objectives, which can be formalized in terms of statistical loss functions [12]. For parameter estimation, one usually minimizes the trace of the expected posterior matrix (cf. so-called “A-optimality” [13]). For model selection, one chooses the input *u* that minimizes the so-called “Laplace-Chernoff risk” 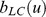, which is an analytical approximation to the model selection error rate [14]. For example, with two models and assuming that (i) both models are a priori equally likely, and (ii) both prior predictive densities have similar variances 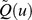, the Laplace-Chernoff risk is given by:
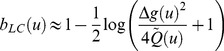
(6)where 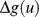 is the difference in the first-order moment of the data predictions under model 1 and model 2, respectively (cf. Figure 3). Optimizing 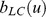 with respect to the design *u* thus reduces to discriminating the predictions under the candidate models, either by increasing the distance between their first-order moments, and/or by decreasing their second-order moments 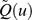. The latter derives from the gradient of the observation function with respect to model parameters.

Critically, optimizing the classical efficiency of the design (i.e. statistical power) minimizes the Laplace-Chernoff risk for the equivalent Bayesian model comparison. This is important, since it allows one to generalise established experimental design rules to a data analysis. We refer the interested reader to [14].

Critically, the optimality of the design relates to the experimental question. In our example, the best design for assessing whether effort devaluates incentive value may not be the same as the best design for identifying the precise way in which this devaluation occurs. In addition, one might want to exploit the real-time accumulation of information to perform on-line design optimization. The toolbox is equipped to address such problems (see, e.g., section “Dynamic causal modelling of fMRI data” below).

### Organization of the toolbox

The toolbox is organized as follows:

- The root folder (/DAVB) contains a core set of (matlab) routines that implement the VB approach to stochastic nonlinear state-space models [15]. The main inversion routine is ‘VBA_NLStateSpaceModel.m’, which is described in more details below. The root folder also contains group-level inference schemes (cf. ‘VBA_groupBMS.m’, [16]) and post-hoc model selection tools (cf. ‘VBA_SavageDickey.m’, [17]), as well as routines for model simulation (cf. ‘simulateNLSS.m’) and results visualization (cf. ‘VBA_ReDisplay.m’, ‘VBA_PPM.m’). Functions that perform post-hoc inversion diagnostics (such as Volterra kernel estimation, cf. ‘VBA_VolterraKernels.m’) are stored in this folder. Finally, this folder gathers routines that can be used to evaluate and optimize the efficiency of the experimental design, with respect to either model comparison or parameter estimation (cf. ‘VBA_designEfficiency.m’, [14]).
- The subfolder ‘/DAVB/stats&plots’ contains non-specific routines that deal with either statistical and/or display issues, example of which include general linear model and classical contrast inference (‘GLM_contrast.m’), 3D visualization of time-dependant probability density functions (‘plotDensity.m’), or receiver operating characteristic analysis (‘doROC.m’)…
- The subfolder ‘/DAVB/subfunctions’ contains all sorts of example evolution and observation functions, as well as demonstration scripts. A selection of the latter will be described in the next section. This is where demonstration scripts, as well as evolution and observation functions of models for behaviour and neuroimaging data are stored (see examples in section “Results” below).

From a practical viewpoint, inserting a model into the toolbox only requires the specification of the observation and evolution functions (see below). In the next section, we will highlight a few example applications, in order to demonstrate the capabilities of the toolbox. Note that a link to an interactive graphical summary of the toolbox can be found on the toolbox's internet wiki pages (http://code.google.com/p/mbb-vb-toolbox/).

### Summary of the input/output format of the main functions

Most importantly, evolution and observation functions have to conform to a standardized I/O form: *[fx,dfdx,dfdP] = fname(x,P,u,in)*, where:

- *x*: current hidden state value
- *P*: evolution/observation parameter value
- *u*: current input value
- *in*: this is an arbitrary variable that can contain any additional information that has to be passed to evolution/observation functions
- *fx*: the current evaluation of the evolution/observation function
- *dfdx/dfdP*: these are optional output arguments, which quantify the gradients of the evolution/observation function w.r.t. to hidden states and parameters, respectively. The main inversion routine automatically detects whether these are returned by the evolution/observation function (if not, numerical derivation is used internally).

In addition, the main inversion routine (‘VBA_NLStateSpaceModel.m’) has the following I/O form: *[posterior,out] = VBA_NLStateSpaceModel(y,u,f_fname,g_fname,dim,options)*, where:

- *y/u*: these are the observed data and controlled input to the system (see above).
- *f_fname* (resp. *g_fname*): name/handle of the function that returns the evolution (resp. observation) of the hidden states.
- *dim*: a structure variable containing the dimensions of the 3 sets of the model's unknown variables (*n*, *n_theta* and *n_phi*).
- *options*: an optional structure containing specific information regarding the model, i.e.: prior sufficient statistics on model parameters, mircrotime resolution (see below), additional information that may have to be passed to evolution/observation functions, lag *k* for the VBA-Kalman forward pass, VB convergence variables (e.g. maximum number of iterations, minimum increment in free energy), delay matrix (see below), flag for continuous/categorical data (see below), etc…
- *posterior*: a structure variable whose fields contain the sufficient statistics (typically first and second order moments) of the VB approximation to the posterior densities over the observation/evolution/precision parameters and hidden-states time series.
- *out*: a structure variable that recapitulates the optional arguments (in case defaults were used) and provides supplementary information regarding the VB model inversion (e.g., model diagnostics: free energy, percentage of variance explained, Volterra kernels, etc…).

We refer the interested reader to the header of ‘VBA_NLStateSpaceModel.m’, as well as to example scripts described in the next section. In addition to matlab workspace variables, the main inversion routine returns a graphical summary of the VB inversion (this can be retrieved using *VBA_ReDisplay(posterior,out)*). This summary is organized in tabs, which are described on the toolbox's wiki page (http://code.google.com/p/mbb-vb-toolbox/wiki/Results).

## Results

We will first expose a couple of examples of standard models for behavioural and neuroimaging data, respectively. This will serve to demonstrate the capabilities of the toolbox. We will then focus on a few special cases of the broad class of generative models defined above. We believe these examples deserve special attention, given their relevance in the context of models for behavioural and neurobiological data. Finally, we will focus on the more specific issues of (i) performing model selection at the group level (given that the best model may differ across subjects), and (ii) using data-driven stochastic system identification together with inversion diagnostics to constrain and/or improve computational models of neurobiological and/or behavioural data.

### Subject-level analyses: Examples

#### Reinforcement learning models of choice data

In psychological terms, motivation can be defined as the set of processes that generate goals and thus determine behaviour. A goal is nothing else than a “state of affairs”, to which people attribute (subjective) value. Empirically speaking, one can access these values by many means, including subjective verbal report or decision making. Biological signals such as vegetative responses (e.g., skin conductance or pupil dilation) have also been linked to value, through its effect on arousal [18]. These measures have been used to demonstrate how value changes as people learn a new operant response [19]. This is predicted by reinforcement learning theories, which essentially relate behavioural response frequency to reward [20]. In this context, value is expected reward, and it changes in proportion to the agent's prediction error, i.e. the difference between actual and expected reward [21]. In the form of Equation 1 (first line), this learning rule can be captured by the following evolution function (in this case, the system is deterministic, cf. section “What about deterministic systems?”):
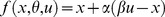
(7)where *x* denotes value, *u* is the environmental feedback and *α* is the learning rate. In this notation, experienced reward depends upon the environmental feedback through some arbitrary scaling parameter *β*. For example, one may consider the possibility of some asymmetry in the relative weight of, e.g., financial gains and losses [22]. This could be captured by letting *β* depend upon whether the feedback is positive (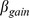) or negative (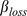). Alternatively, one may assume that asymmetry in the behavioural response to gains and losses may be due to different learning rates, (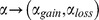).

The script ‘demo_asymRW.m’ simulates an experiment that aims at discriminating between these hypotheses. We consider a go/no-go choice design, whereby the (artificial) subject decides, at each trial, whether to gamble or not. On the one hand, if he gambles (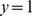), he receives either a positive, negative or neutral feedback (e.g., 1€, −1€, 0€). On the other hand, if he chooses the ‘no-go’ option (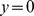), he receives nothing (neutral feedback). The decision to gamble or not can be thought as an economic choice, which is driven by the learned value of the ‘go’ option. Here, we model the likelihood 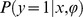 of choosing the ‘go’ option using the following softmax mapping of the ‘go’ value *x*:
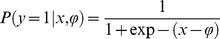
(8)where 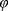 captures a potential bias toward the ‘no-go’ option. Mathematical details regarding the definition of such categorical (Bernouilli) likelihood function are given in section “Handling categorical observations” below. Learning occurs after each ‘go’ trial, given the choice outcome *u* (cf. equation 7). In this example, the feedbacks *u* for each ‘go’ trial were randomly sampled following the relative frequencies: 2/5 positive, 2/5 negative, 1/5 neutral.

**Figure 4:**
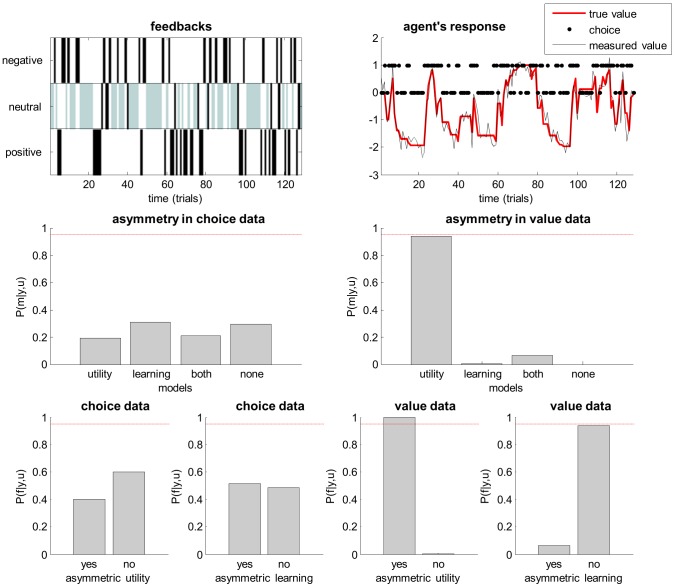
Comparison of asymmetric utility and asymmetric learning rate. This figure summarizes the analysis of choice and value data using models that assume asymmetric utility, asymmetric learning rate, both asymmetries or none. **Upper left**: Trial-by-trial feedback history (either negative, neutral or positive). Grey neutral feedbacks correspond to ‘no-go’ choices. **Upper right**: Trial-by-trial dynamics of true value (red), measured value (black) and agent's binary go(1)/no-go(0) choices (black dots). **Middle-left**: posterior probability of the four models given simulated choice data. **Middle-right**: same format, given value data. **Lower left**: family posterior probabilities for both model spaces partitions, given choice data (left: family ‘yes’ = {‘utility’, ‘both’} vs family ‘no’ = {‘learning’, ‘none’}, right: family ‘yes’ = {‘learning’, ‘both’} vs family ‘no’ = {‘utility’, none’}. **Lower left**: same format, given value data.

We then performed a Bayesian model comparison of four models (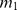: asymmetric utility, 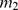: asymmetric learning, 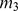: both types of asymmetry, and 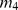: no asymmetry), given either observed choices (categorical data) or vegetative responses (continuous data). Here, the latter type of data is simply simulated by adding random noise to the true value time series (SNR = 1 dB). The ensuing likelihood function has the Gaussian form given in the second line of Equation 1, where the observation function *g* has been set to the identity mapping. Importantly, both types of data are simulated under model 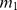. Figure 4 summarizes the results of this simulation.

Not surprisingly, one can see that the value data is much more informative than the choice data. Overall, there is hardly any statistical evidence in favour of any form of asymmetry in the choice data. In contradistinction, Bayesian model comparison based upon value data correctly identifies the presence of asymmetry in the experienced reward (model ‘asymmetric utility’). We also performed family-inference, which consists in partitioning model space into subsets of models [23]. Here, we chose two orthogonal partitions, which induce two pairs of model families: (i) 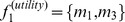 versus 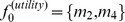, and (ii) 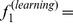

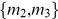
 versus 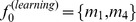. The first (resp. second) family comparison pools evidence for or against utility (resp. learning) asymmetry. Results of the family comparisons confirm the model comparisons, demonstrating that no dimension of the model space is strongly informed by choice data. In contradistinction, family inference given continuous value data (correctly) provides evidence for utility asymmetry, and against learning asymmetry.

#### Dynamic causal modelling of fMRI data

Decomposing the relation existing between cognitive functions and their neurobiological “signature” (the spatio-temporal properties of brain activity) requires an understanding of how information is transmitted through brain networks [24]. The ambition here is to ask questions such as: “what is the nature of the information that region A passes on to region B”? This stems from the notion of functional integration [25], which views function as an emergent property of brain networks. Dynamic causal modelling –DCM- has been specifically developed to address this question (see the seminal DCM work in [26], and a recent review in [5]). In DCM, hemodynamic (fMRI) signals arise from a network of functionally segregated sources; i.e., brain regions or neuronal sources. First, DCM describes how experimental manipulations (*u*) influence the dynamics of hidden neuronal states of the system (*x*). This is typically written in the form of Equation 1 (first line), where the neural evolution function is given by [27]:
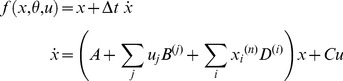
(9)where 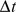 is the discretization time step, and the parameters *θ* of this neural evolution function include a between-region coupling (matrix *A*), input-dependent coupling modulation (matrices 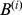), input driving gains (matrix *C*) and gating effects (matrices 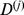). DCM also includes the effect of the hemodynamic response function, which effectively performs a temporal convolution operation on neural states [28].

**Figure 5:**
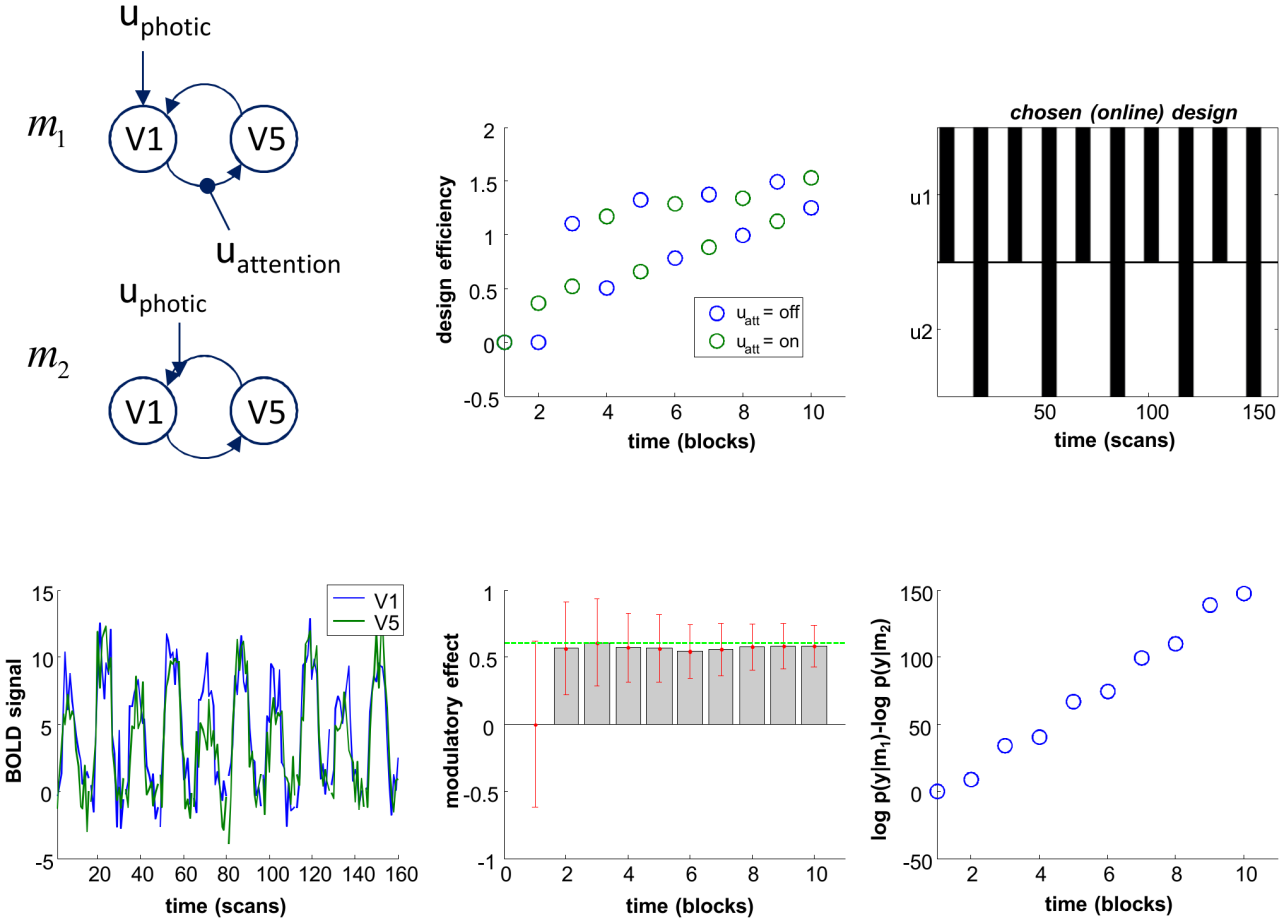
Online design optimization for DCM comparison. This figure summarizes the simulation of online design optimization, in the aim of best discriminating between two brain network models (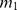 and 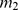) given fMRI data time series. In this case, the problem reduces to deciding whether or not to introduce the second experimental factor (here, 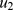 = attentional modulation), on top of the first factor (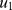 = photic stimulation). **Upper left**: the two network models to be compared given fMRI data (top/bottom: with/without attentional modulation of the V1→V5 connection). **Upper middle**: block-by-block temporal dynamics of design efficiency of both types of blocks. Green (resp. blue) dots correspond to blocks with (resp. without.) attentional modulation. **Upper right**: scan-by-scan temporal dynamics of the optimized (on-line) design. **Lower left**: scan-by-scan temporal dynamics of the simulated fMRI signal (blue: V1, green: V5). **Lower middle**: block-by-block temporal dynamics of 95% posterior confidence intervals on the estimated modulatory effect (under model 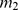). The green line depicts the strength of the simulated effect. **Lower right**: block-by-block temporal dynamics of log Bayes factors 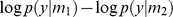.

An exhaustive assessment of the properties of DCM simulation and VB model inversion can be found elsewhere [29]–[30], [12]. In this section, we will focus on the issue of online design optimization. We refer the interested reader to the script ‘demo_dcmonline.m’. This demo simplifies the network identification of [26]. In brief, photic input enters V1, which is reciprocally connected to V5. The goal of the experiment is to assess whether attention modulates the feedforward connection from V1 to V5. This is addressed using Bayesian model comparison given fMRI data (i.e. model 1: attention modulates the V1→V5 connection; model 2: no modulatory effect). Within the session, each block consists of 16 seconds of stimulation and 16 of rest. The on-line design optimization consists in deciding, before each block, whether we modulate subjects' attention. This is done by comparing the design efficiency (i.e. –minus- the Laplace-Chernoff risk that is induced by the comparison of the two models) of two canonical block designs, i.e. photic stimulation with and without attentional modulation. Then, the fMRI response to the chosen stimulation is simulated under the true (but unknown) model. Critically, the evaluation of the design efficiency changes as the experiment unfolds, because both models are inverted given each new block dataset, yielding increasingly precise model predictions. In brief, the on-line design optimization procedure implements the experimental cycle of Figure 1, keeping the set of alternative hypotheses unchanged. Figure 5 summarizes the results of this on-line design optimization.

First, one can see that the design efficiency increases with time, as we expected. Second, the most efficient design alternates across blocks. Eventually, the best design (identified by the on-line procedure) is such that an attentional manipulation is performed each two blocks. It turns out that this is the maximally orthogonal design (correlation between u_photic_ and u_attention_ = 0.6), under the constraint that photic stimulation is always on. Interestingly, this reproduces the design chosen in the original fMRI experimental study [31]. In addition, one can see that the posterior credible interval of the attentional modulatory effect converges toward the true (simulated) value. Lastly, the log Bayes factor (as derived from VB free energies) increases with time. This indicates that there is increasing evidence in favour of the true model, as the experiment unfolds.

### Special cases

#### What about deterministic systems?

Deterministic systems can be understood as a particular case of stochastic nonlinear state-space models. They arise at the infinite state noise precision limit (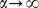). Such limit follow from setting the Gamma priors accordingly, i.e. 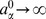 and 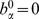. The hidden states trajectory *x* then becomes an implicit function of the evolution parameters 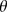, the initial conditions 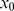 and the system's input 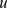 (if any). This implies that the identification of such deterministic systems can be treated like a static model (see below) of the form 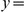

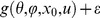
. This simply means that, holding the evolution/observation parameters and initial conditions fixed, one invariably obtains the exact same deterministic predictions of the experimental data (up to measurement errors or residuals).

Note that the VB treatment of stochastic state-space models is systematically initialized by identifying the deterministic variant of the dynamical system. We invite the reader interested in the comparison of stochastic and deterministic model inversions to run demos such as ‘demo_Lorenz.m’, ‘demo_VanDerPol.m’ or ‘demo_doubleWell.m’, which reproduce the exemplar analyses in [15].

**Figure 6:**
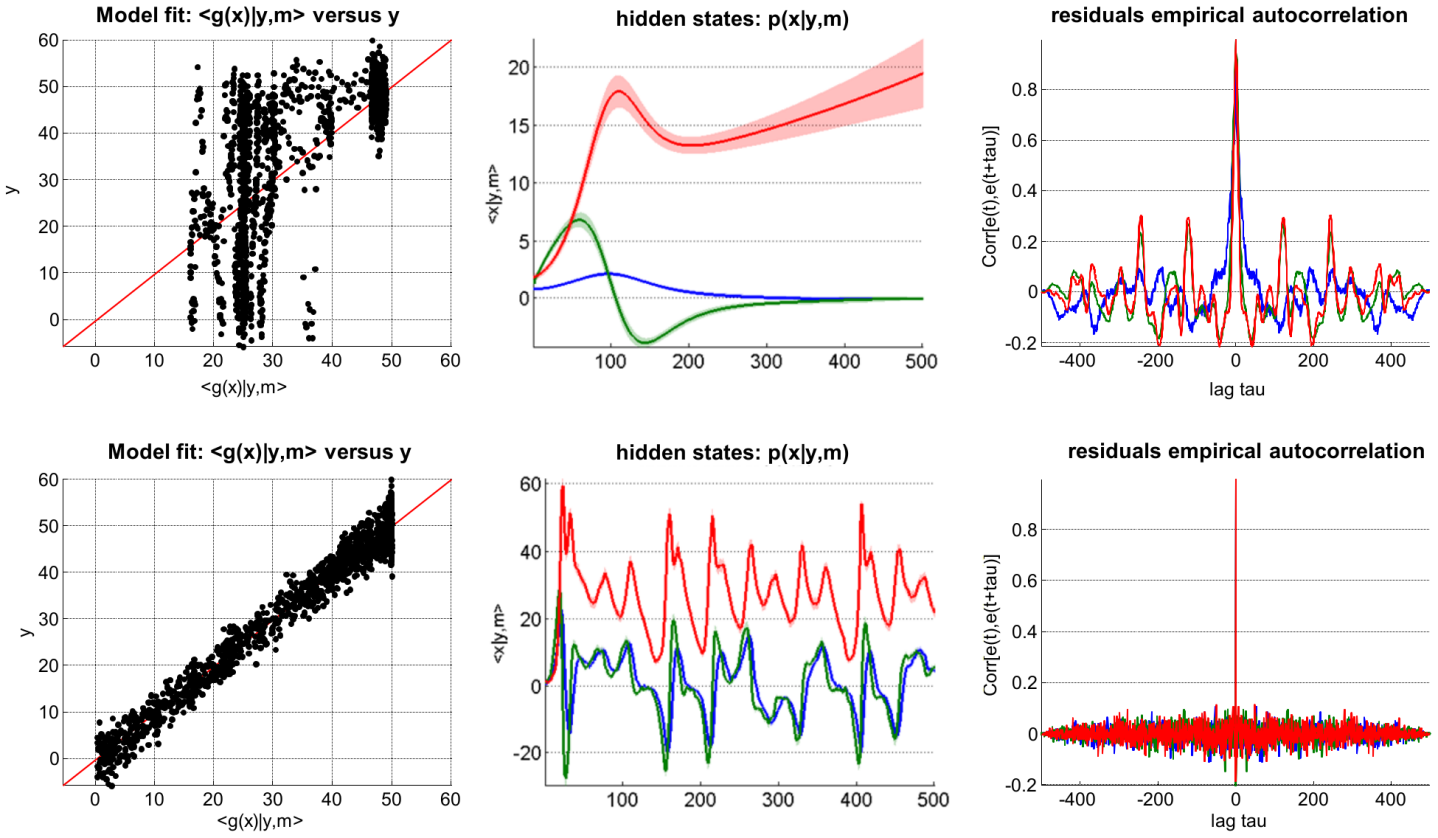
Comparison of deterministic and stochastic dynamical systems. This figure summarizes the VB comparison of deterministic (upper row) and stochastic (lower row) variants of a Lorenz dynamical system, given data simulated under the stochastic variant of the model. **Upper left**: fitted data (x-axis) is plotted against simulated data (y-axis), for the deterministic case. Perfect model fit would align all points on the red line. **Lower left**: same format, for the stochastic case. **Upper middle**: 95% posterior confidence intervals on hidden-states dynamics. Recall that for deterministic systems, uncertainty in the hidden states arises from evolution parameters' uncertainty. **Lower middle**: same format, stochastic system. **Upper right**: residuals' empirical autocorrelation (y-axis) as a function of temporal lag (x-axis), for the deterministic system. **Lower right**: same format, stochastic system.

Figure 6 summarizes the comparison of the VB inversion of deterministic and stochastic variants of the Lorenz system (this Figure can be reproduced from the script ‘demo_Lorenz.m’). One can see the profound impact of state noise, both on hidden states trajectories and on the structure of model residuals (estimated measurement noise). More precisely, model inversion under the ‘no state-noise’ assumption leaves a lot of unexplained variance in the data. Critically, model residuals exhibit strong temporal structure (cf. lobes in the temporal autocorrelation). In addition, the magnitude of model residuals seems to depend upon the model predictions. Such structured model residuals typically signal *underfitting*. In contradistinction, the stochastic model inversion produces residuals that have no particular structure (they seem to be equally distributed around model predictions and have no temporal autocorrelation). In this example, Bayesian model comparison based upon the VB free energy correctly favours the stochastic model.

#### Dealing with delays

Delayed dynamical systems are generally non-Markovian, in their native form. Consider, for example, a system whose evolution is given by: 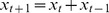 (cf. sequence of Fibonacci numbers). Here, one needs to know both its current and delayed (one step back in time) state to predict the system's next state. VBA can deal with certain forms of delays by embedding the state space *x* into an augmented (Markovian) state-space *X*, where 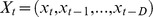. Here, *D* is the maximum delay considered. The dimension of the embedded state space is now 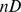, which can be considerably high. The script ‘demo_delays.m’ provides a demonstration of the inversion of a delayed stochastic dynamical system. The demo first sets up priors and optional arguments, which include the delay matrix *D*. In this case, the system is a two-dimensional linear system with (delayed) feedback.

**Figure 7:**
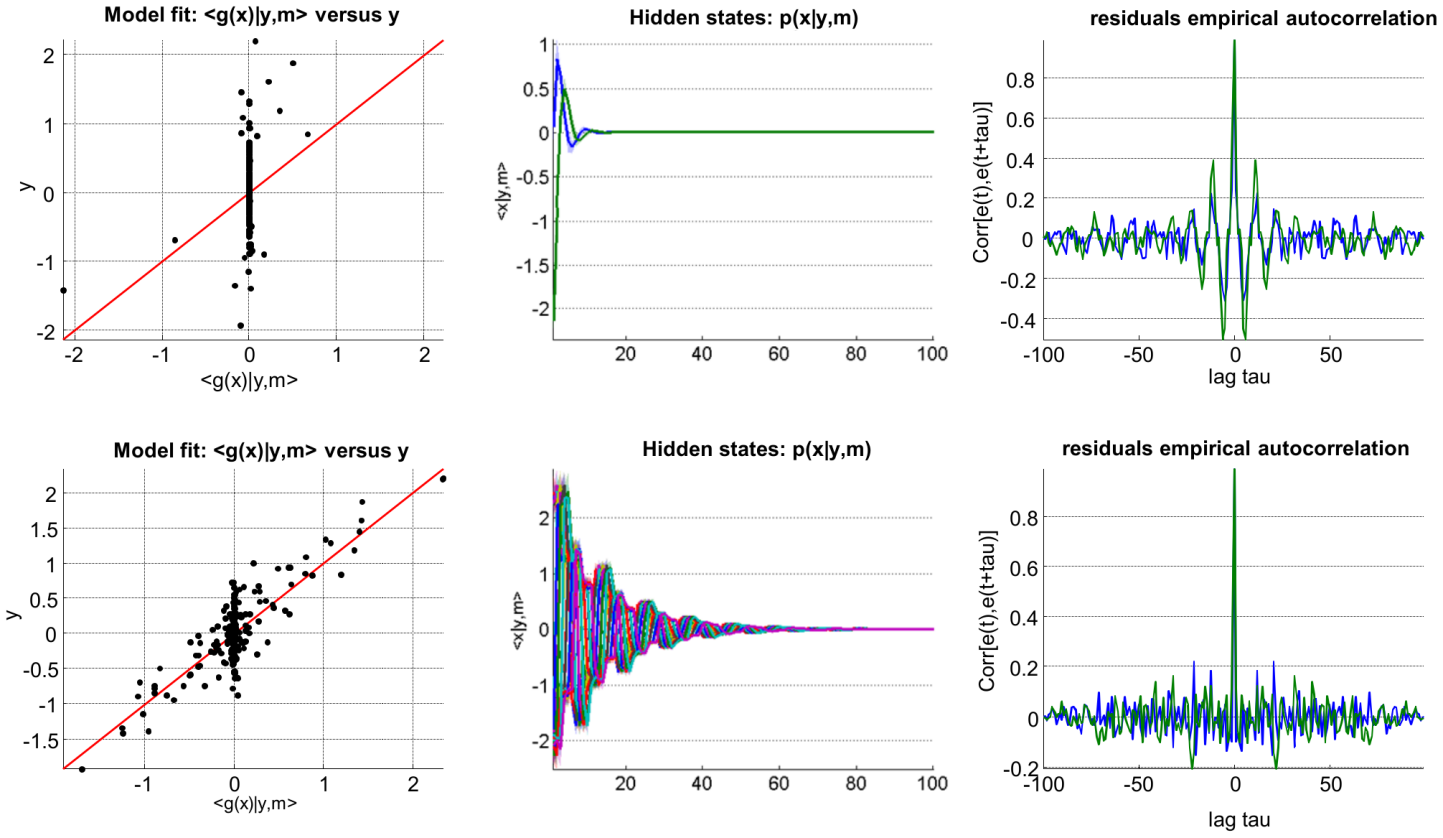
Comparison of delayed and non-delayed dynamical systems. This figure summarizes the VB comparison of non-delayed (upper row) and delayed (lower row) variants of a linear deterministic dynamical system, given data simulated under the delayed variant of the model. This figure uses the same format as Figure 6.

Figure 7 summarizes the comparison of the ensuing VB inversions of (deterministic) delayed and non-delayed variants of the system. One can see the profound impact of delays, both on hidden states trajectories and on the structure of model residuals. In addition, the incorrect (non-delayed) model yields residuals with striking structure (cf. model fit and temporal autocorrelation). In comparison, the delayed (correct) model inversion displays rather weak residual structure. Here again, VB model comparison favours the correct model.

Alternatively, one may construct a corrected evolution function, using a first-order Taylor expansion around zero-delays. We refer the interested reader to the Text S1 in the supplementary information (see also the appendix in [32]).

#### Considering serially correlated noise

The toolbox can deal with most forms of auto-regressive or state-dependant noises (both at the level of hidden states and observations). It suffices to construct an augmented state space *X*, where 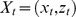, and appropriately modify the evolution and observation functions, as well as the priors. For example, the evolution of a stochastic system driven with AR(1) noise could be modelled as follows:
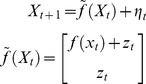
(10)where *f* is the evolution function on the original state space *x*, and 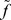 is its modification on the augmented state space *X*. Importantly, under Equation 6, both AR(1) and white noise (respectively 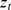 and 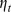) can drive the system. To ensure that *z* is the most likely driving force, one can set the augmented state noise (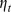) covariance matrix 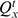, such that its upper-left half block is close to zero. In addition, one may have to increase the lag *k*. This is because the effect of the AR(1) delayed state noise on the hidden states is maximal one sample ahead in time. On thus need to look one step back in time to infer on the delayed state noise.

**Figure 8:**
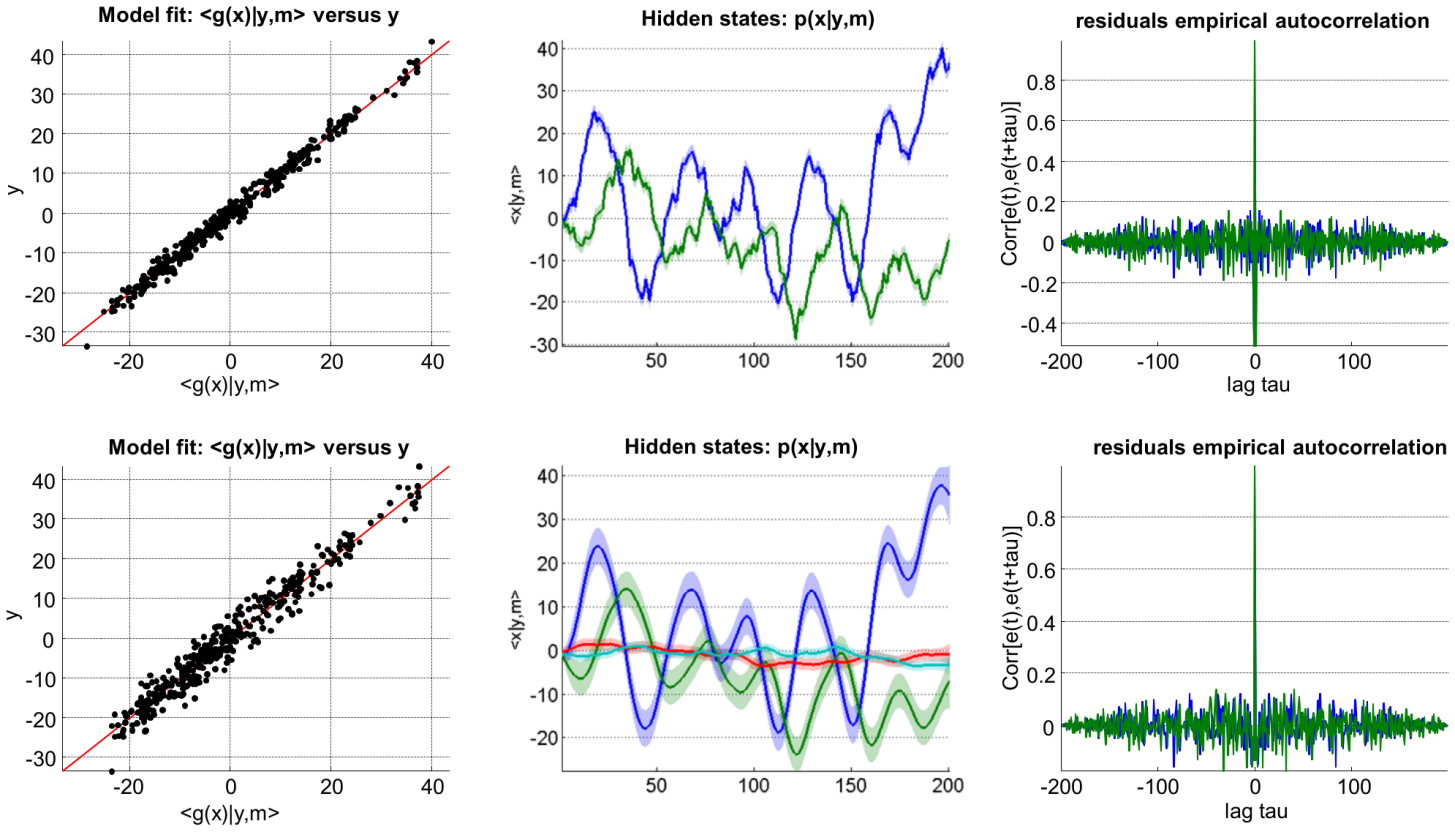
Comparison of white and auto-correlated state-noise. This figure summarizes the VB comparison of stochastic systems driven with either white (upper row) or auto-correlated (lower row) state noise. This figure uses the same format as Figure 6.

Figure 8 summarizes the comparison of the VB inversion of a simple linear stochastic system with AR(1) state noise, having assumed AR(1) noise or not (cf. script ‘demo_AR1.m’). One can see the impact of correlation in state noise, both on hidden states trajectories and on the structure of model residuals. More precisely, residuals of the ‘white state-noise’ model exhibit a clear (negative) peak in their autocorrelation function, at lag one. This is due to the model's inability to capture AR(1) state noise. In contradistinction, the AR(1) model inversion show no temporal structure in its residuals. Interestingly, the AR(1) model yields residuals with a higher magnitude than the “white state-noise” model. This is because the AR(1) model imposed (correct) smoothness constraints on hidden states trajectories. This eventually prevented the model from *overfitting* the data, which seems to have occurred for the ‘white state-noise’ model.

Autoregressive processes of any order can be obtained by augmenting the state space with delayed state noise 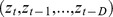, where *D* is the order of the autoregressive process. This is basically using the above delay embedding trick (and requires an appropriate increase of the lag *k*). In fact, almost all forms of state-dependent noise can be modeled using essentially the same strategy. It suffices to replace the last term in the right-hand side of equation 10 (second line) with any non-linear function of the augmented states (e.g.: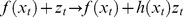, where 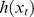 plays the role of the state-dependent noise standard deviation).

#### Getting closer to continuous dynamical systems

The evolution equation of Equation 1 is discrete in time. If one directly uses this as an approximation to a continuous dynamical system, then the approximation's accuracy strongly depends on the data sampling frequency (c.f. Text S3 in the supplementary information). However, the toolbox allows one to specify a “microtime” resolution, which is used to recursively apply the evolution function between two time samples. This can be useful to control the time discretization errors introduced when approximating the original continuous dynamical system. For example, let us assume that the system obeys the following ODE: 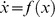. We wish to derive a prediction from 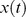 to the next time sample, 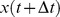, where 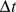 is the time lag between two data samples. One can do this by recursively applying the time discretization scheme on a smaller time grid. For example, if one uses an Euler discretization scheme (cf. Text S3 in the supplementary information):
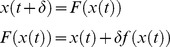
(11)where *δ* is a small integration step (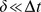). Then the recursive application of the Euler evolution function *F* yields:
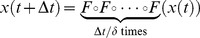
(12)where the function *F* is evaluated recursively (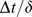 times) between two time samples. Here, 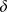 induces a “microtime” resolution grid. Note that this procedure does not allow state noise to enter anywhere else than at the times where data are sampled. This means that the impact of state noise will be somehow underestimated. However, in the context of deterministic systems, increasing the microtime resolution eventually yields very accurate approximations to continuous dynamics.

**Figure 9:**
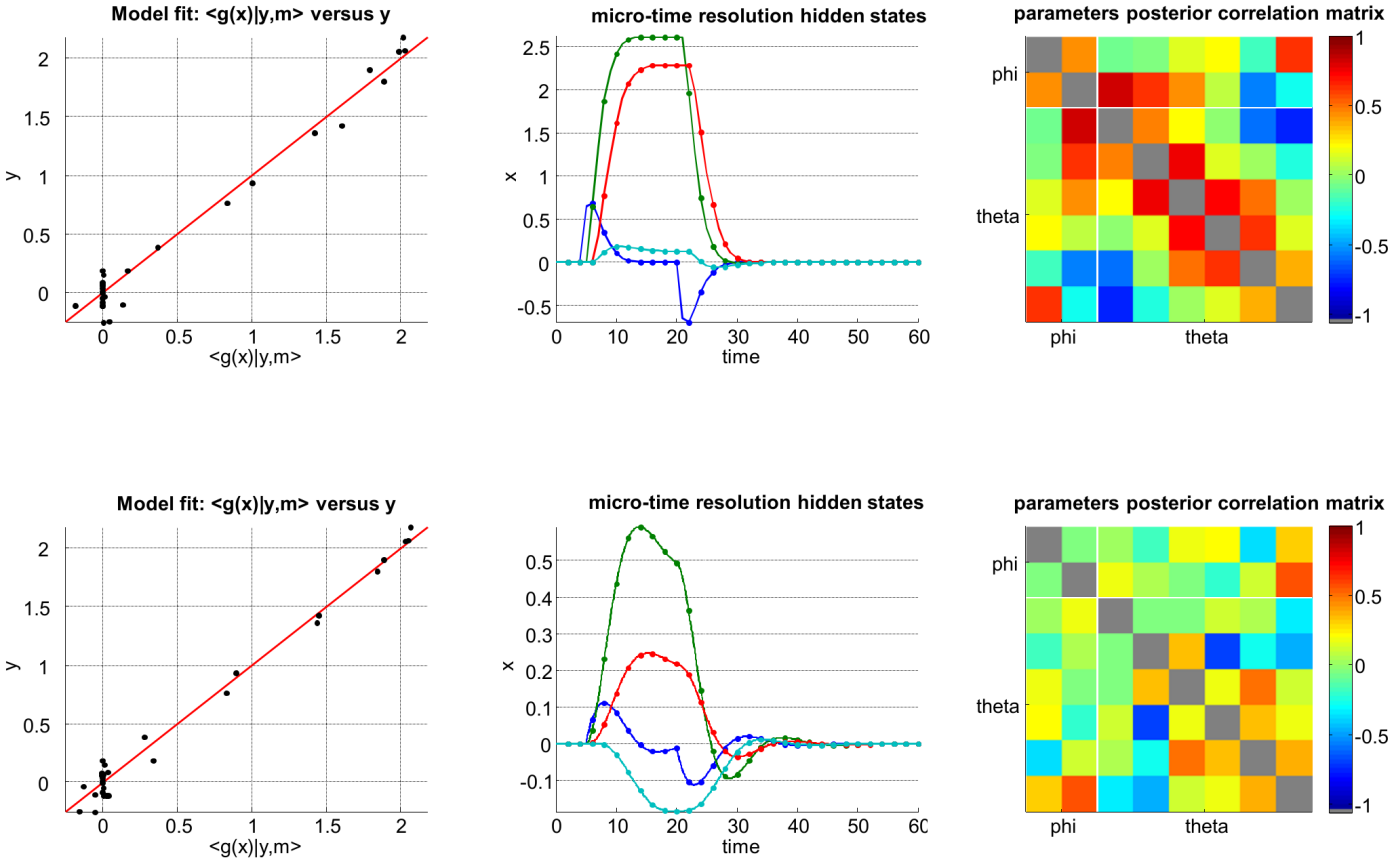
Effect of the micro-time resolution. This figure summarizes the effect of relying on either a slow (upper row) or fast (lower row) micro-time resolution, when inverting nonlinear dynamical systems. **Left**: same format as Figure 6. **Upper middle**: estimated hidden-states dynamics at low micro-time resolution (data samples are depicted using dots). **Lower middle**: same format, fast micro-time resolution. **Upper right**: parameters' posterior correlation matrix, at low micro-time resolution. **Lower middle**: same format, fast micro-time resolution.

Figure 9 summarizes the impact of reducing the microtime resolution on the VB inversion of a model of the hemodynamic response function (cf. script ‘demo_HRF.m’). In this example, the structure of model residuals does not discriminate between the two variants of the model. However, one can see how different the estimated states' trajectories are. In addition, the impact of unknown model parameters on the model predictions depends upon the microtime resolution. This can be seen in the structure of the posterior correlation matrix. In this particular example, decreasing the microtime resolution eventually compromises parameter identifiability. In other words, the information that can be retrieved on the evolution/observation parameters is degraded when reducing the microtime resolution.

#### Handling categorical observations

Strictly speaking, the generative model of Equations 1–3 copes with continuous data, for which there is a natural distance metric. Now if the data is categorical, there is no such natural metric, and one has to resort to probability distributions dealing with discrete events. For example, binary data can be treated as binomial (Bernouilli) samples, whose sufficient statistic (first-order moment) is given by the observation function. This means that the observation equation (second line of Equation 1) is replaced by the following binomial likelihood function:
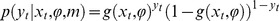
(13)where 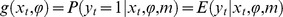, by definition. Here, the measurement noise precision 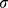 and covariance components 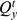 are irrelevant. It turns out that this does not induce any major change in the VB inversion scheme under the Laplace approximation. We refer the interested reader to the Text S2 in the supplementary information. In fact, when the dimension of the data is high enough (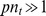), the empirical distribution of the ‘residuals’ 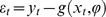 will tend to a Gaussian density. This means that one can then approximate the above likelihood with a Gaussian density with mean 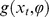 and (unknown) precision 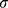.

An interesting application is binary data classification, which can be understood as a special case of Equation 13. In the linear case, the observation mapping reduces to a linear mixture passed through a sigmoid mapping, i.e.: 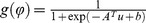, where *u* is an arbitrary vector of explanatory variables (i.e. features), and *A* (resp. *b*) is an unknown vector (resp. scalar) that encodes the linear mapping (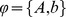).

**Figure 10:**
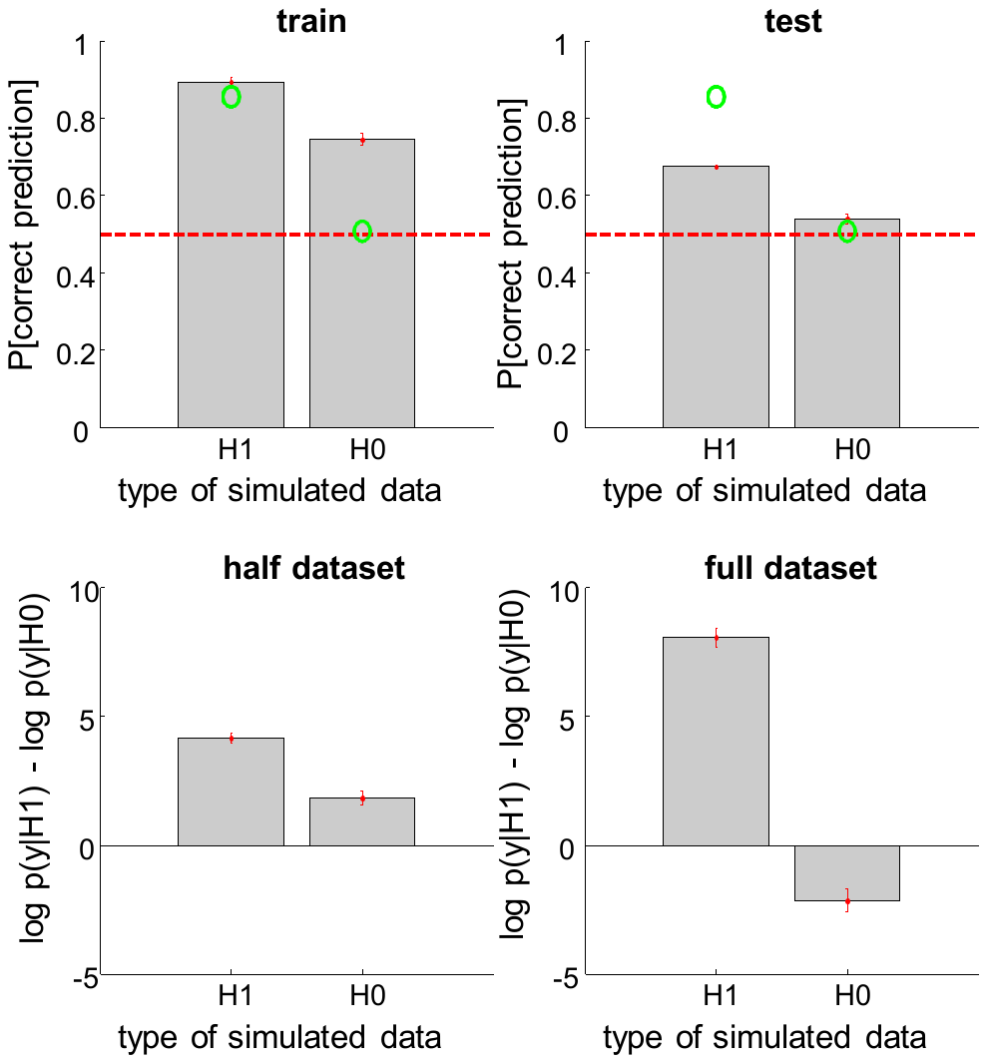
Binary data classification. This figure exemplifies a classification analysis, which is used to infer on the link between a continuous variable *X* and a binary data *y*. The analysis is conducted on data simulated under either a null model (H0: no link) or a sigmoid mapping (H1). **Upper left**: the classification accuracy, in terms of the Monte-Carlo average probability of correct prediction under both types of data (left: H1, right: H0), for the training dataset. The green dots show the expected classification accuracy, using the true values of each model's set of parameters. The dotted red line depicts chance level. **Upper right**: same format, test dataset (no model fitting). **Lower left**: same format, for the log Bayes factor 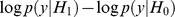, given the training dataset. **Lower right**: same format, given the full (train+test) dataset.

Figure 10 (script ‘demo_bin.m’) summarizes the statistical performance of the VB approach to data classification. In brief, we have simulated categorical data under two different models, namely: H0 (no systematic link between features *u* and data *y*, i.e. 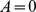) and H1 (which posits an arbitrary but non-zero mapping *A*). Both H0 and H1 were then inverted given either the first half (to perform classical cross-validation on test-data) or the full dataset (to perform Bayesian model comparison). This procedure was repeated 256 times, in order to derive average performance measures.

One can see how Bayesian model comparison replicates the generalizability measure of standard cross-validation procedures, provided the inference is based upon the whole dataset (rather than splat into two train/test halves). More precisely, one can see that classification performance reaches statistical significance when the data are simulated under H1, but not under H0. This falls from the predictive density over ‘test’ data obtained after the VB inversion of the H1 model given ‘train’ data. Equivalently, VB model comparison of H1 and H0 correctly identifies the true model, with greater confidence when the whole dataset is used for deriving the free energy.

#### Inverting static (hierarchical) models

Static models are simplifications of the above class of generative models, where the dimension of the hidden states and the evolution parameters tend to zero (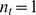, 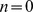 and 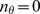). In this case, the generative model reduces to a nonlinear observation equation, i.e.: 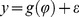, with fixed priors on the observation parameters.

Alternatively, a simple (two-levels) hierarchical extension of this static model (whereby the priors are also learned) can easily be derived by actually removing the evolution/observation parameters, but retaining the initial conditions and the first hidden states, with, e.g., identity evolution function, i.e.:
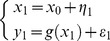
(14)where 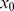, 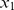, 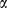 and 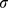 are estimated. This is the typical structure for so-called “mixed-effects” models for analysis at the group level. Critically, estimating the “initial conditions” 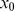 as well as the “state noise” precision 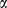 enables one to infer on the group mean and variance.

**Figure 11:**
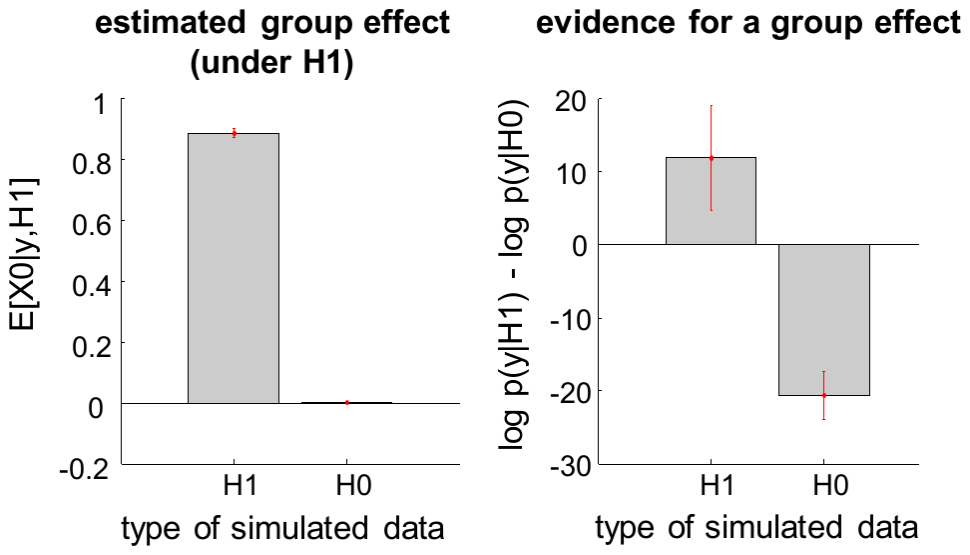
Random-effect analysis. This figure exemplifies a random-effect GLM analysis, which is used to infer on the group mean of an effect of interest. The analysis is conducted on data simulated under either a null model (H0: group mean is zero) or a non-zero RFX model (H1). **Left**: Monte-Carlo average of the VB-estimated group mean under H1, given both types of data (left: H1, right: H0). **Left**: same format, for the log Bayes factor 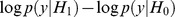.

Figure 11 (script ‘demo_RFX.m’) summarizes the statistical performance of the VB treatment of mixed-effect models (cf. Equation 14). One can see that both the estimated group mean and the Bayesian model comparison are coherent, in terms of inferring whether there is a non-zero group-mean (second level effect). More precisely, the posterior estimate of the group mean is different from zero when the data are simulated under H1 (which posits an arbitrary but non-zero group mean), but not under H0 (zero group mean). Equivalently, the VB model comparison correctly identifies H1 from H0, across Monte-Carlo simulations.

### Group-level Bayesian model selection

The issue of performing random effects Bayesian model selection (BMS) at the group level was originally addressed in [16]. In this work, models were treated as random effects that could differ between subjects and have a fixed (unknown) distribution in the population. Here, this hierarchical model is inverted using a VB scheme, to provide conditional estimates of the frequency with which any model prevails in the population. This random effects BMS procedure complements fixed effects procedures that assume subjects are sampled from a homogenous population with one (unknown) model (cf. the log group Bayes factor that sums log-evidences over subjects). [16] also introduced the notion of exceedance probability, which measures how likely it is that any given model is more frequent than all other models in the comparison set. These two summary statistics typically constitute the results of random effects BMS (see, e.g., [33]). In addition, the toolbox also returns the model attributions, i.e. the posterior probability, for each subject, of being best described by each model.

**Figure 12:**
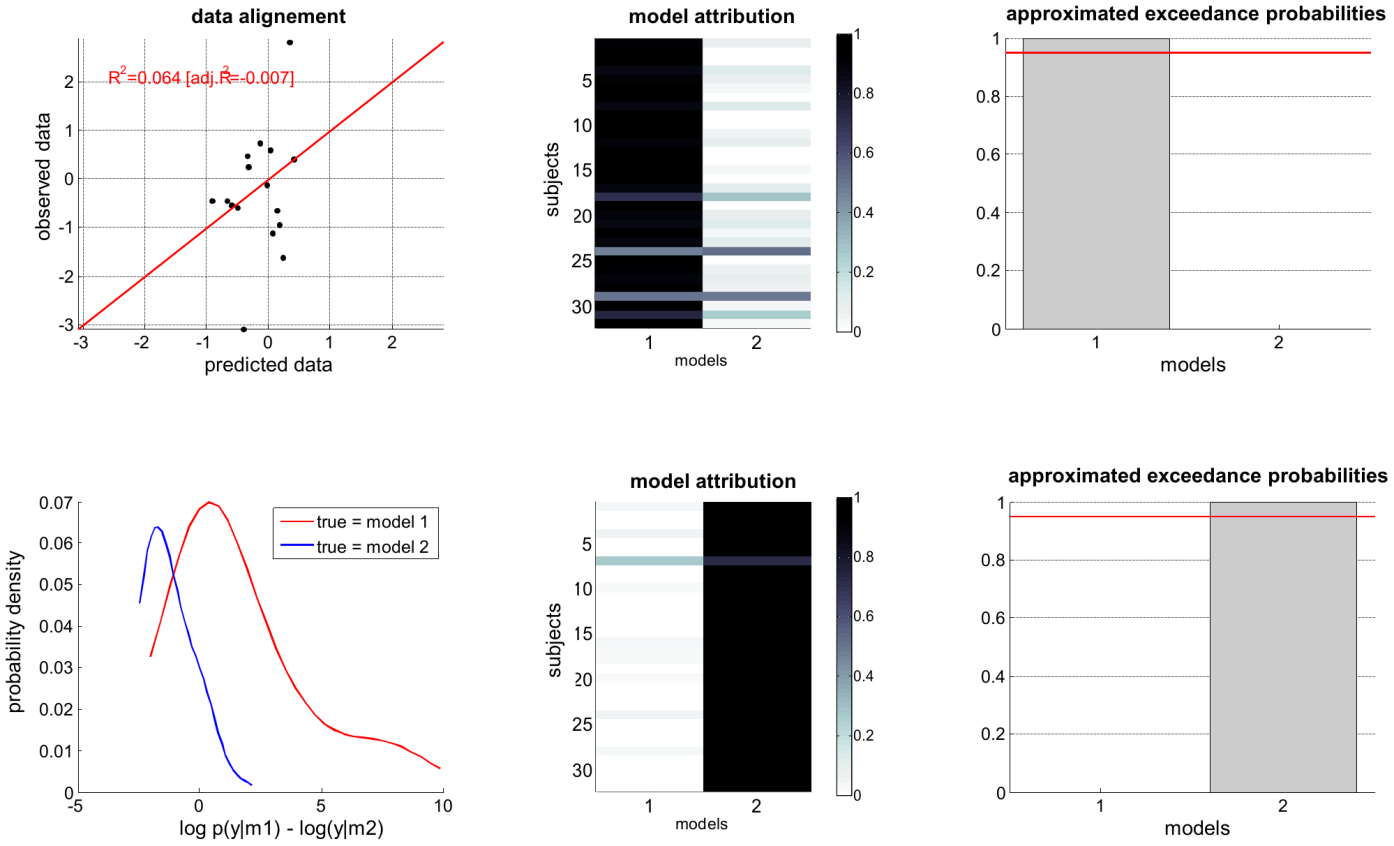
Random-effect group-BMS. This figure exemplifies a random-effect group-BMS analysis, which is used to infer on the best model at the group level. The analysis is conducted on two groups of 32 subjects, whose data were simulated under either a ‘full’ (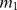, group 1) or a ‘reduced’ (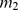, group 2) model. **Upper left**: simulated data (y-axis) plotted against fitted data (x-axis), for a typical simulation. **Lower left**: histograms of log Bayes factor 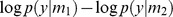, for both groups (red: group 1, blue: group 2). **Upper middle**: model attributions, for group 1. The posterior probability 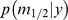 for each subject is coded on a black-and-white colour scale (black = 1, white = 0). **Lower middle**: same format, group 2. **Upper right**: exceedance probabilities, for group 1. The red line indicates the usual 95% threshold. **Lower right**: same format, group 2.

Figure 12 (script ‘demo_bmc4glm.m’) demonstrates the random-effect group BMS approach, in the context of a simple static general linear model (GLM). In brief, we simulated two groups of 32 subjects (under arbitrary subject-specific GLMs), one of which only expressing half of the (four) factors. This allows us to derive the Monte-Carlo distribution of within-subjects' data under both a ‘full’ and a ‘reduced’ model. One can see the quality of the fit for a typical simulated dataset (SNR = 0 dB). The log-model evidence of both models was derived for each data (here, at the frequentist limit, cf. ‘lev_GLM.m’). First, observe that the Monte-Carlo histograms of the log-Bayes factors under each model are only partially separated. This is due to the amount of measurement noise. However, despite the relatively weak identifiability of the two models, the model attributions exhibit almost no uncertainty, and the exceedance probabilities clearly identify the underlying model.

### Improving computational models using inversion diagnostics

Identifying relevant mechanisms is arguably the most difficult task in modelling complex behavioural and/or biological data. In fact, one may not in a position to suggest an informed model for the data before the experiment. For example, when modelling how subjects update the value of alternative options given the feedback they receive, one may assume that the learning rate may change over trials. However, one may not know what the determinants of learning rate adaptation are. A practical solution to this problem is to first treat the learning rate as a stochastic hidden state, whose random walk dynamics cannot be *a priori* predicted. One would then use the VBA inversion of such a model to estimate the learning rate dynamics from, e.g., observed people's choices. Finally, one could then perform a Volterra decomposition of hidden states dynamics onto a set of appropriately chosen basis functions. This diagnostic analysis allows one to identify the hidden states' impulse response to experimentally controlled inputs to the system. We refer the interested reader to Text S3 in the supplementary information for mathematical details regarding Volterra decompositions.

**Figure 13:**
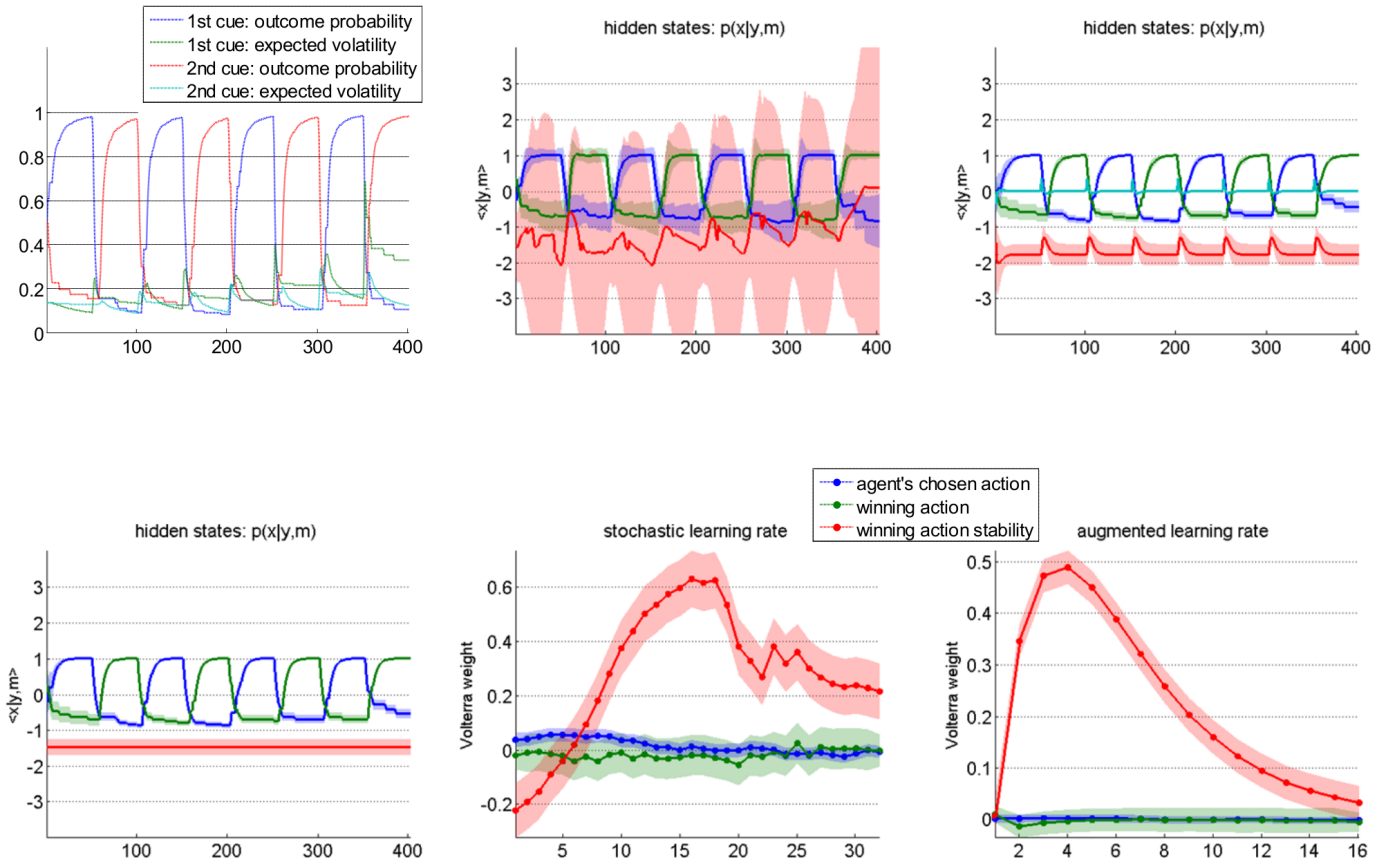
Improving Q-learning models with inversion diagnostics. This figure demonstrates the added-value of Volterra decompositions, when deriving learning models with changing learning rates. **Upper left**: simulated belief (blue/red: outcome probability for the first/second action, green/magenta: volatility of the outcome contingency for the first/second action) of the Bayesian volatile learner (y-axis) plotted against trials (x-axis). **Lower left**: estimated hidden states of the deterministic variant of the dynamic learning rate model (blue/green: first/second action value, red: learning rate). This model corresponds to the standard Q-learning model (the learning rate is constant over time). **Upper middle**: estimated hidden states of the stochastic variant of the dynamic learning rate model (same format). Note the wide posterior uncertainty around the learning rate estimates. **Lower middle**: Volterra decomposition of the stochastic learning rate (blue: agent's chosen action, green: winning action, red: winning action instability). **Upper right**: estimated hidden states of the augmented Q-learning model (same format as before). **Lower right**: Volterra decomposition of the augmented Q-learning model's learning rate (same format as before).

Figure 13 (script “demo_dynLearningRate.m”) demonstrates the above procedure, in the context of the two-armed bandit problem [34]. In brief, an agent has to choose between two alternative actions, each of which may yield positive or negative feedbacks. In our case, we reversed the action-outcome contingency every fifty trials. First, a series of choices are simulated, under an agent model that learns both the evolving action-outcome probabilities and their volatility [7]. The agent's inferred volatility increases after each contingency reversal, and then decays back to steady-state (cf. upper-left panel in Figure 13). The VBA toolbox is then used to invert a “dynamical” variant of the Q-learning model (cf. section “Reinforcement learning models of choice data”), given the agent's sequence of choices. More precisely, the state-space was augmented with the learning rate, whose state-noise precision was set hundred time smaller than that of action values. This is to ensure that stochastic deviations from deterministic learning dynamics originate from changes in learning rate. One can see (cf. lower-left panel in Figure 13) that the estimated learning rate dynamics strongly correlates with the simulated agent's inferred volatility (classical test: F = 176.1, p<10^−8^). The ensuing Volterra decomposition was performed w.r.t. three input basis functions, namely: the agent's chosen action, the winning action (which might not be the chosen action), and the winning action instability. The latter input is one when the winning action has changed between the previous and the current trial, and is zero otherwise. The Q-learner's choice behaviour is driven by the difference in action values, which mainly responds to the history of winning actions (not shown). In contradistinction, the learning rate exhibits a stereotypical response to winning action instability (cf. middle panels in Figure 13). This diagnosis can then be used to augment Q-learning models with deterministic learning rate dynamics, whose impulse response mimic the estimated Volterra kernel. An example of the evolution function of such an augmented Q-learning model is given by (cf. Text S3 in the supplementary information):
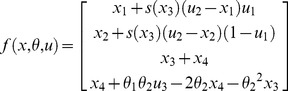
(15)where *s* is the sigmoid mapping, 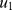 is the agent's previous choice, 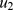 is the previous feedback, 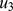 is the winning action instability, 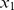 (resp. 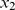) is the value of the first (resp. second) action, 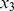 is the (inverse-sigmoid transformed) learning rate and 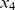 is its discrete temporal derivative. Here, 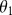 weighs the impact of 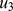 onto the learning rate, and 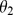 controls the decay rate of the impulse response. Such augmented Q-learning model predicts a transient acceleration of the learning rate following changes in the winning action whenever 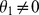. This is confirmed by the VBA inversion of this model (cf. right panels in Figure 13). Finally, Bayesian model comparison yields overwhelming evidence in favour of the augmented Q-learning model, when compared to the standard Q-learning model (VBA free energies were: 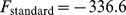 and 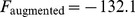).

This concludes the demonstration of the VBA toolbox.

## Availability and Future Directions

In this paper, we have exposed the main algorithmic components of the VBA toolbox, which implements a probabilistic treatment of nonlinear models for neurobiological and behavioural data. This toolbox aims at disseminating models and methods that serve experimental purposes, and providing a flexible platform, which modellers can easily contribute to.

VBA is under intense development as we speak. More precisely, the following additions to the current toolbox's version are under test:

- Between-conditions and between-groups second-level Bayesian model selection. This rests on quantifying the evidence for a difference in model labels (resp. frequencies) across conditions (resp. groups). We refer the interested reader to [35].
- Dual categorical/continuous data analysis. In particular, this is necessary for inverting models that aim at explaining concurrent neuroimaging time series and trial-by-trial behavioural observations such as choices.
- VB inversion of mixture models (e.g., mixtures of gaussians and binomials). The objective here is to handle data-driven probabilistic clustering approaches, that can serve as reference points for model-based data analyses.
- Extension of the nonlinear state-space model to arbitrary variance components. This is most useful when dealing with data pooled from qualitatively different sources with potentially very different SNRs (e.g., neuroimaging and skin conductance time series).
- Higher-level functionalities that allow to handle multiple sessions, parameter mappings (e.g., for positivity constraints), factorial family partitioning of model space, etc … The need for such extensions increases as the diversity of VBA users' interests steadily grows.
- Library of observation/evolution functions of models for behavioural and neuroimaging data. These include, but are not limited to: learning rules (e.g., bayesian belief updates with different forms of priors [5]–7, deterministic exploration, cognitive dissonance effects [36], …), canonical utility functions (e.g., delay discounting [37], effort devaluation [38], risk attitude, …), neural spiking dynamics (e.g. Hodgkin-Huxley [39], Fitz-Hugh-Nagumo [40], …), neural meso-scale networks (e.g., Jansen-Ritt [41], neural fields [42], …), etc…

Some of these extensions are already available from the current code distribution. We encourage the interested reader to look for appropriate key words in the demonstration scripts.

VBA's code is under open-source GNU General Public Licence (v2), and is freely downloadable from the toolbox's internet wiki pages (http://code.google.com/p/mbb-vb-toolbox/wiki/InstallingTheToolbox). These wiki pages expose a lot of user-oriented information, as well as detailed examples and screen captures. The wiki also serves to gather comments, criticism, suggestions and contribution of VBA users.

## Supporting Information

Software S1 VBA source code. Note that an interactive graphical summary of the toolbox can be found on the toolbox's internet wiki pages (http://code.google.com/p/mbb-vb-toolbox/).
(ZIP)

Text S1 Dealing with unknown delays in systems' dynamics.
(DOCX)

Text S2 VB-Laplace inversion of models for categorical (binary) data.
(DOCX)

Text S3 Mathematical details regarding the relationship between integral and differential forms, the transition from continuous to discrete time formulations and Volterra decompositions of systems' dynamics.
(DOCX)
