# Supplementary material for: VBA: A Probabilistic Treatment of Nonlinear Models for Neurobiological and Behavioural Data
Source: PLoS Comput Biol. 2014 Jan 23;10(1):e1003441. doi: 10.1371/journal.pcbi.1003441 (PMC3900378; doi:10.1371/journal.pcbi.1003441)
Supplement: Text S1 — Dealing with unknown delays in systems' dynamics. (DOCX) [file pcbi.1003441.s002.docx]

Dynamical systems: dealing with unknown delays

This note complements the section "Dealing with delays" of the main manuscript. The idea we develop here is construct a *corrected* evolution function, using a first-order Taylor expansion around zero-delays (see, e.g., [32]).

Let us assume that the system obeys the following ODE (in continuous time):

$\frac{dx}{dt}=f\left(x\left(t-D\right)\right)$ (A1)

Now let us Taylor-expand the evolution function around $D=0$:

$f\left(x\left(t-D\right)\right)\approx f\left(x\left(t\right)\right)-D\frac{df}{dx}\frac{dx}{dt}$ (A2)

Replacing Equation A2 into Equation A1 now yields:

$\frac{dx}{dt}=\left[I+D\frac{df}{dx}\right]^{-1}f\left(x\left(t\right)\right)$ (A3)

which can then be used to derive a "delay-corrected" evolution function in discrete time. For example, an Euler discretization scheme now yields:

$x_{t+1}=x_{t}+\Delta t\left[I+D\frac{df}{dx}\right]^{-1}f\left(x_{t}\right)+\eta_{t}$ (A4)

where $\Delta t$ is the time discretization step and the state noise $\eta_{t}$ lumps together $O\left(D^{2}\right)$ error terms and any other perturbations. Note that in this formulation, delays can now be estimated as part of the evolution parameters ($D\in\theta$).

The VBA toolbox does not (yet) automatically perform such analytical approximation of delayed dynamics. This means that one has to implement Equation A4 directly in the system's evolution function.
