# Supplementary material for: VBA: A Probabilistic Treatment of Nonlinear Models for Neurobiological and Behavioural Data
Source: PLoS Comput Biol. 2014 Jan 23;10(1):e1003441. doi: 10.1371/journal.pcbi.1003441 (PMC3900378; doi:10.1371/journal.pcbi.1003441)
Supplement: Text S2 — VB-Laplace inversion of models for categorical (binary) data. (DOCX) [file pcbi.1003441.s003.docx]

VB approximate inference: dealing with categorical observations

First, let us consider a simple generative model (with no hidden states) dealing with binary data variables. Without loss of generality, the likelihood function can be written as the following Bernouilli conditional distribution:

$\begin{matrix} p\left(y\left|\vartheta,m\right.\right)=\prod_{t=1}^{n_{t}} p\left(y_{t}\left|\vartheta,m\right.\right) \\ p\left(y_{t}\left|\vartheta,m\right.\right)=g_{t}\left(\vartheta\right)^{y_{t}}\left(1-g_{t}\left(\vartheta\right)\right)^{1-y_{t}} \end{matrix}$ (A5)

where $y_{t}$ is a binary data point (there are $n_{t}$ of these), whose first-order moment $g_{t}\left(\vartheta\right)$ is determined by model parameters, i.e.: $p\left(y_{t}=1\left|\vartheta,m\right.\right)=\left\langle y_{t}\left|\vartheta,m\right.\right\rangle=g_{t}\left(\vartheta\right)$. By analogy with Equations 1-3 in the main text, we will refer to the function $g$ as the observation mapping.

Given priors $p\left(\vartheta\left|m\right.\right)$ on model parameters, any arbitrary probability density function $q\left(\vartheta\right)$ induces a free-energy bound on the log-model evidence:

$\begin{matrix} F=\left\langle I\left(\vartheta\right)\right\rangle_{q\left(\vartheta\right)}+S\left(q\left(\vartheta\right)\right) \\ \approx I\left(\mu\right)+\frac{1}{2}\ln\left|\Sigma\right|+\frac{n_{\vartheta}}{2}\ln2\pi\\ I\left(\vartheta\right)=\log p\left(\vartheta\left|m\right.\right)+\sum_{t=1}^{n_{t}} y_{t}\log g_{t}\left(\vartheta\right)+\left(1-y_{t}\right)\log\left(1-g_{t}\left(\vartheta\right)\right) \end{matrix}$ (A6)

where we have used the Laplace approximation, yielding a Gaussian fixed-form approximate posterior density $q\left(\vartheta\right)=N\left(\mu,\Sigma\right)$. Under Normal priors ($p\left(\vartheta\left|m\right.\right)=N\left(\mu_{0},\Sigma_{0}\right)$), the gradient and Hessian of the log- joint density $I\left(\vartheta\right)$ then write:

$\begin{matrix} \frac{\partial I}{\partial\vartheta}=\Sigma_{0}^{-1}\left(\mu_{0}-\theta\right)+\sum_{t=1}^{n_{t}} \frac{y_{t}-g_{t}\left(\vartheta\right)}{g_{t}\left(\vartheta\right)\left(1-g_{t}\left(\vartheta\right)\right)}\frac{\partial g_{t}}{\partial\vartheta} \\ \frac{\partial^{2}I}{\partial\vartheta^{2}}\approx-\Sigma_{0}^{-1}-\sum_{t=1}^{n_{t}} \left(\frac{y_{t}}{g_{t}\left(\vartheta\right)^{2}}+\frac{1-y_{t}}{\left(1-g_{t}\left(\vartheta\right)\right)^{2}}\right)\frac{\partial g_{t}}{\partial\vartheta}^{T}\frac{\partial g_{t}}{\partial\vartheta} \end{matrix}$ (A7)

where the second-order derivatives of the observation mapping have been neglected. This is actually necessary in the general case, because it can potentially induce non positive-definite covariance matrices. This yields an analytical form for the second-order moment of the approximate (Laplace) posterior density:

$\Sigma\approx\left[\Sigma_{0}^{-1}+\frac{\partial g}{\partial\vartheta}^{T}Diag\left(\frac{y}{g\left(\vartheta\right)^{2}}+\frac{1-y}{\left(1-g\left(\vartheta\right)\right)^{2}}\right)\frac{\partial g}{\partial\vartheta}\right]^{-1}$ (A8)

where the divide and square operators within the brackets of the right-hand-side of Equation A8 improperly denote the element-by-element operations on the appropriate vectors.

From here, the extension to a nonlinear stochastic state-space model is almost trivial. One simply needs to replace the log-density log- joint density $I\left(\vartheta\right)$ by its expectation over the appropriate model variable partition. This is what the VBA toolbox automatically does when *options.binomial* is set to 1.

Note that when dealing with categorical (binomial) observations, the precision $\sigma$ of model residuals becomes irrelevant. However, one can still eyeball estimated model residuals $\hat{e}=y-g\left(\hat{x}\right)$ and their autocorrelation function from the "diagnostics" tab (cf. "VBA_ReDisplay.m"). Although these residuals are now bounded between -1 and 1, their interpretation is similar to the continuous (gaussian) case.
