# Supplementary material for: VBA: A Probabilistic Treatment of Nonlinear Models for Neurobiological and Behavioural Data
Source: PLoS Comput Biol. 2014 Jan 23;10(1):e1003441. doi: 10.1371/journal.pcbi.1003441 (PMC3900378; doi:10.1371/journal.pcbi.1003441)
Supplement: Text S3 — Mathematical details regarding the relationship between integral and differential forms, the transition from continuous to discrete time formulations and Volterra decompositions of systems' dynamics. (DOCX) [file pcbi.1003441.s004.docx]

Dynamical systems: integral and differential formulations, transition from continuous to discrete time and Volterra kernels

**Continuous dynamical systems: from integral to differential forms**

Let $x\left(t\right)$ be defined by the following integral equation:

$x\left(t\right)=\int_{-\infty}^{\infty} u\left(t'\right)g\left(t,t'\right)dt'\begin{matrix} \\ \end{matrix}$ (A9)

where $u\left(t\right)$ is an arbitrary function time. If $g$ is an invariant kernel (that is, if $g\left(t,t'\right)=g\left(t-t'\right)$), then the right-hand term of equation A9 becomes a convolution operation and $g$ is simply the impulse response function of $x\left(t\right)$(to inputs $u\left(t\right)$). In this case, one can derive a differential form of Eq. A9. For example, let $g$ be given by the truncated (causal) exponential kernel:

$g\left(t\right)=\left\{\begin{matrix} e^{-at}\text{ if }t\geq0 \\ 0\text{ otherwise} \end{matrix}\right.$ (A10)

Applying the Fourier transform on the left and right hand sides of equation A9 then yields:

$\begin{matrix} \tilde{x}\left(\omega\right)=\tilde{u}\left(\omega\right)\tilde{g}\left(\omega\right) \\ =\frac{\tilde{u}\left(\omega\right)}{a+2\pi i\omega} \Rightarrow\\ 2\pi i\omega\tilde{x}\left(\omega\right)=\tilde{u}\left(\omega\right)-a\tilde{x}\left(\omega\right) \end{matrix}$ (A11)

where the tilde notation refers to the Fourier-transformed functions of time and $\omega$ is the Fourier dual variable (frequency). Applying the inverse Fourier transform on the left and right hand sides of equation A11 now gives the following differential form for Eqs. A9-10:

(A12)

where the dot operator refers to the temporal derivative $\partial/\partial t$. Other forms of the convolution kernel basically yield different ordinary differential equations (cf. Table A1).

| impulse response function | ODE |
| --- | --- |
| $g\left(t\right)=\left\{\begin{matrix} \gamma ate^{-at}\text{ if }t\geq0 \\ 0\text{ otherwise} \end{matrix}\right.$ |  |
| $g\left(t\right)=e^{-\left\vert at\right\vert}$ |  |
| $g\left(t\right)=\frac{t}{\left\vert t\right\vert}$ |  |

**Table A1**. Examples of ODEs induced by different impulse response functions.

When integrated over time (cf. next section), ODEs yield the temporal dynamics of $x\left(t\right)$, given inputs $u\left(t\right)$. Importantly, convolution and ODE formulations are exactly equivalent. In particular, this implies that the instantaneous relationship between the motion of $x\left(t\right)$ and the inputs $u\left(t\right)$ (implicit in the ODE formulation) captures the fact that $x\left(t\right)$ is actually dependent upon the history of past inputs (implicit in the convolution formulation). We have used this equivalence in the last section of the manuscript, when deriving the "augmented" Q-learning model (cf. first line of Table A1).

**From continuous to discrete dynamical systems**

Most continuous dynamical systems can be described using ODEs, with the following normal form:

(A13)

where $x$ are the system's states, $u$ are some arbitrary exogenous inputs that drive the system's states, and $f$ returns the system's motion given its current state $x$. The ODE in Equation A13 can also be written using the following integral formulation:

$x\left(t+\Delta t\right)=x\left(t\right)+\int_{t}^{t+\Delta t} f\left(x\left(t'\right),u\left(t'\right)\right) dt'\begin{matrix} \\ \end{matrix}$ (A14)

where $\Delta t$ is the discretization step. Note that Equation A14 may not be a convolution operation. Equation A14 can be converted into a finite difference equation using local linearization, as follows:

$\begin{matrix} x_{t+1}=F\left(x_{t},u_{t}\right) \\ F\left(x_{t},u_{t}\right)\approx x_{t}+J^{-1}\left(\exp\left[J\Delta t\right]-I\right)f\left(x_{t},u_{t}\right) \end{matrix}$ (A15)

where $t$ now indexes discrete time steps, $I$ is the identity matrix and $J=\partial f/\partial x$ is the system's Jacobian (evaluated at the current state $x_{t}$). Critically, when the discretization step $\Delta t$ is small compared to the inverse of the highest eigenvalue of $J$, one has: . Inserting this limit into Eq. A15 yields the so-called Euler discretization scheme, which is accurate whenever $\Delta t$ is orders of magnitude smaller than the quickest decay time of the system. We have used this in the section "Getting closer to continuous dynamical systems" of the main manuscript, when introducing the "microtime resolution" of dynamical systems in the VBA toolbox.

**Volterra decomposition**

Most dynamical systems can be described in terms of input-output relationships, where the output $x_{t}$ is typically a function of the history of past inputs $u_{t-1},u_{t-2},...,u_{t-\tau}$ to the system. Volterra series allow a decomposition of the input-output transformation, as follows:

$x_{t}=\omega^{(0)}+\sum_{\tau} \omega_{\tau}^{(1)}u_{t-\tau}+\sum_{\tau_{1}} \sum_{\tau_{2}} \omega_{\tau_{1},\tau_{2}}^{(2)}u_{t-\tau_{1}}u_{t-\tau_{2}}+...$ (A16)

where we have chosen a discrete-time formulation. Here, $\tau$ is some arbitrary time lag and we have truncated the series (up to second order). Equation A16 essentially derives from a recursive Taylor expansion of Equation A14. First-order Volterra kernels $\omega^{(1)}$ capture the linear weight of lagged inputs onto the output. First-order Volterra kernels would be equivalent to impulse response functions, would the system be linear (cf. Equation A9 in continuous time). Second-order Volterra kernels $\omega^{(2)}$ capture additional hysteresis effects, whereby the systematic response to some past input depends upon the input it received at some other point in time. For example, systems exhibiting "refractory periods" can be captured using second-order Volterra kernels that cancel the first-order impact of inputs during recovery time.

When performing dynamical systems' inversion, the VBA toolbox computes the first-order Volterra kernels of the sampled system's observable outputs and estimated hidden states and observables, w.r.t. to all inputs. The estimation is performed by the function ‘VBA_VolterraKernels.m’, which essentially uses the VBA inversion of Equation A16, given some maximum lag (set in *options.kernelSize*; default=16). Note that one can perform a Volterra decomposition of the system w.r.t. any arbitrary set of inputs by calling ‘VBA_VolterraKernels.m’, having appropriately reset the inputs in *out.u*. Replacing the estimated kernels in *out.diagnostics.kernels* allows one to eyeball the new Volterra decomposition from the "kernels" tab (cf. ‘VBA_ReDisplay.m’). Importantly, fit accuracy metrics (coefficient of determination in the continuous case, and balanced classification accuracy in the binomial case) allows one to evaluate whether the truncated Volterra series faithfully captures the input-output relationship.
